# Can indocyanine green during laparoscopic sleeve gastrectomy be considered a new intraoperative modality for leak testing?

**DOI:** 10.1186/s12893-022-01796-5

**Published:** 2022-09-16

**Authors:** Giovanna Pavone, Alberto Fersini, Mario Pacilli, Michele De Fazio, Piercarmine Panzera, Antonio Ambrosi, Nicola Tartaglia

**Affiliations:** 1 Department of Medical and Surgical Sciences, University of Foggia, Viale Pinto 1, 71122 Foggia, Italy; 2 Department of General Surgery and Liver Transplantation, University of Bari, 70124 Bari, Italy

**Keywords:** Laparoscopic sleeve gastrectomy, Indocyanine green test, Gastric fistula

## Abstract

**Background:**

Indocyanine green (ICG) when injected intravenously into the bloodstream allows us to show stomach vascularity in real time. The aim of our study was to observe the preliminary results of the application of indocyanine green fluorescence (IGF) during laparoscopic sleeve gastrectomy (LSG) in our center and how the perfusion of the staple line of the stomach affects the onset of fistula.

**Materials and methods:**

82 patients underwent LSG with ICG fluorescence angiography at our center from January 2020 to December 2021. 5 ml of ICG was injected intravenously to identify the blood supply of the stomach, carefully assessing the angle of His.

**Results:**

In the ICG-tested LSG, we recorded adequate perfusion in all patients but one: the leakage rate was 1.2%. This data is inferior to the non-tested patients’ group.

**Conclusion:**

Intraoperative ICG testing may be helpful in determining which patients are at an increased risk for leakage but there are multiple factors contribute to the pathophysiology and the incidence of gastric fistula not only the perfusion.

*Trial registration* Retrospectively registrated

## Introduction

Obesity is a rising global epidemic that places significant strain on health care services worldwide. Bariatric surgery has become the most effective way to achieve significant weight loss and improve the associated comorbidities of obesity. Obesity is more prevalent in industrialized countries, and its incidence is continually rising [1].

Laparoscopic sleeve gastrectomy (LSG) has become the most frequently performed bariatric procedure worldwide because compared to Roux-en-Y gastric bypass (BRGY), LSG is less demanding in terms of technical skills required and has a shorter operative time due to the absence of an anastomosis [2].

Gastric leakage is the most serious complication of sleeve gastrectomy.

The pathogenesis of gastric leakage has still not been clarified, even though a multifactorial aetiology (ischaemic, mechanical, inflammatory) has been considered [3–5]. Although there are various methods of detecting intraoperative leaks, no standardized algorithm for the intraoperative diagnosis of gastric leak is available [6–8].

The use of indocyanine green (ICG) has been introduced in minimally invasive surgery [9–11].

Indocyanine green (ICG) is a medical dye that can be injected into the human bloodstream and has no negative side effects or contraindications [12]. ICG fluoresces when exposed to near infrared (NIR)-wavelength light (approximately 820 nm) [13–15]. The fluorescence is detected by special telescopes and is transmitted to a standard monitor that allows for the identification of the anatomical structures in which the dye is present (e.g., biliary tract, vessels, lymph nodes). This is a low-cost technique capable of showing the vascularity of the stomach in real time, and its application to the gastroesophageal (GE) junction during LSG looks very promising [16–18]. The aim of our study was to observe the preliminary results of applying indocyanine green fluorescence (IGF) during laparoscopic sleeve gastrectomy (LSG) in our centre and determine how perfusion of the staple line of the stomach can affect the onset of leakage.

## Materials and methods

Eighty-two consecutive patients underwent LSG with ICG fluorescence angiography at our centre from January 2020 to December 2021.

During this period, 21 male and 61 female patients underwent LSG. The operations were performed by the same surgical team applying the same standardized technique.

The mean age was 43.6 years in the male group and 37.6 years in the female group (mean 39.18 years).

The mean preoperative BMI was comparable between the two groups (45.16 kg/m^2^ in the male group vs. 45.06 kg/m^2^ in the female group).

At least one major comorbidity was found in all patients: the most prevalent was hypertension (52 patients, 63.41%), followed by diabetes (15 patients, 18.3%), COPD and/or OSAS (7 patients, 8.5%), and osteoarthritis (8 patients, 9.7%).

Five patients previously underwent bariatric procedures [endoscopic placement of an IGB (intragastric balloon)] other than bariatric surgery; none of the 82 patients had previous upper gastrointestinal surgery.

After placing four ports, an energy device was used to detach and dissect the short gastric vessels from the greater curvature.

The sleeve resection was shaped around a 40-French bougie held in place using a mechanical linear stapler; 5 ml of ICG was then injected intravenously to identify gastric vascularization, carefully assessing the angle of His [19].

**Fig. 1:**
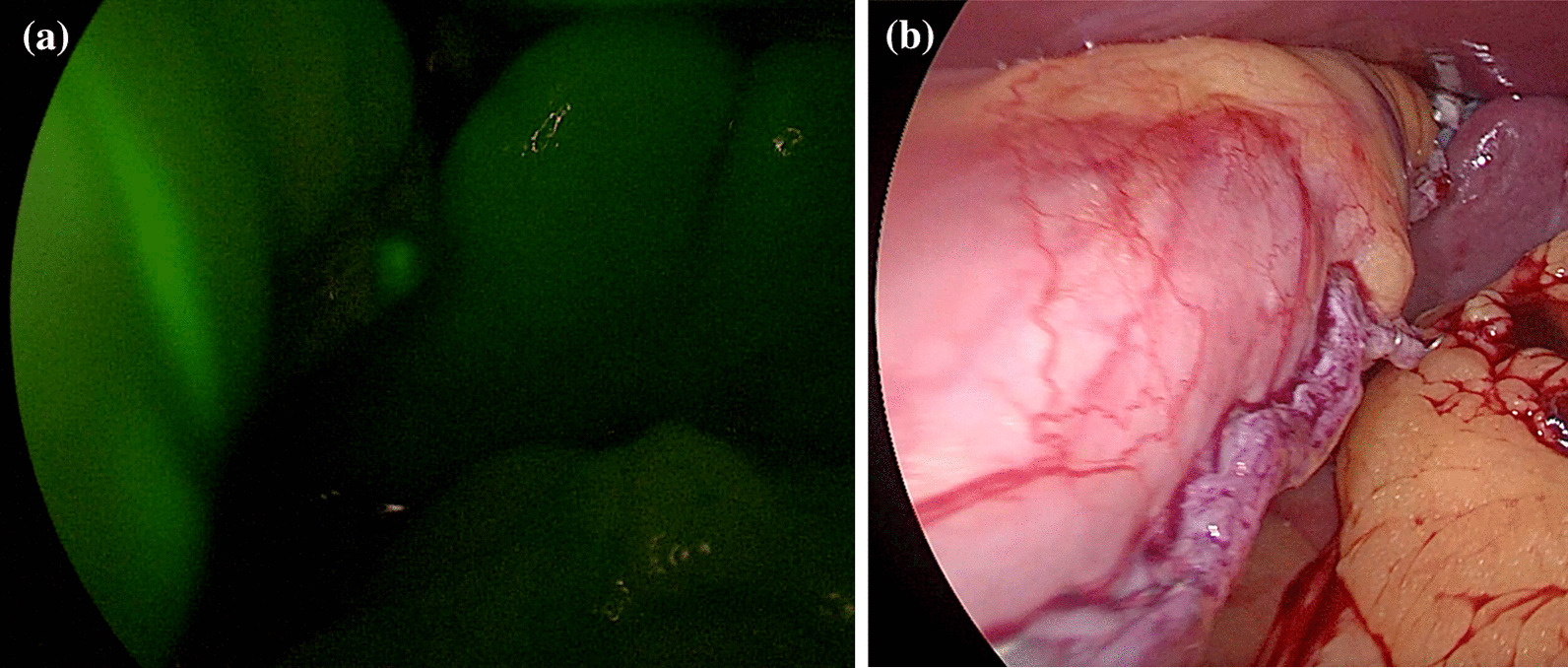
**a** Evaluation of perfusion of the staple line of the stomach by using the indocyanine green test. **b** Intraoperative picture of the staple line of the stomach

Adequate perfusion was defined as “the direct and clear visualization of the fluorescence around the gastric tube, after an estimated time of 150–180 s following i.v. administration” (Fig. 1a, b).

In the event of insufficient perfusion, our anticipated options consisted of suture reinforcement by buttressing (if the gastric tube was large enough at the GE junction and the inadequate perfusion area was confined to the peripheral area) or fibrin glue (if the area of inadequate perfusion was greater than the peripheral area). Intraoperative conversion to Roux-en-Y gastric bypass (RYGBP) was not considered unless perfusion was absent throughout the upper part of the gastric tube (due to the unbearable risk of leakage).

A methylene blue test was routinely performed after fluorescence.

The procedure concluded with the insertion of an intra-abdominal drain along the suture line.

On the second postoperative day, a routine swallow test with Gastrografin was performed.

After a negative Gastrografin swallow test, patients were discharged with a liquid diet for two weeks followed by a semiliquid diet. Follow-up visits routinely comprised blood tests and clinical examination at 3, 6 and 12 months [19].

## Results

The procedure was performed in all patients without ICG-related adverse events.

The methylene blue test was negative in all patients.

Blood supply to the GE junction was rated “satisfactory and adequate” in all patients.

The mean operative time was 53 min. No conversion to laparotomy was performed.

Routine swallow tests with Gastrografin on the second postoperative day were negative for leaks in all patients.

Despite this, one patient (1.2%) showed signs and symptoms related to gastric discharge on the fifth postoperative day, and the diagnosis was confirmed by CT with Gastrografin; she was treated with the endoscopic placement of pigtail stents.

The leakage rate of the group evaluated with ICG was compared with that in the period from 2017 to 2019 in all patients undergoing sleeve gastrectomy in our centre; the total number of patients evaluated without ICG tests who had leakages was 2, with a leakage rate of 2.5%.

## Discussion

Postbariatric surgery leaks remain a serious complication. Several factors have been associated with an increased risk of leakage after LSG [20].

Most gastric leaks (up to 85%) occur near the angle of His at the proximal third of the staple line [8, 21].

The diagnosis of gastrointestinal leak in obese patients after bariatric surgery can be difficult, and such leakages can present with atypical presentations that can lead to catastrophic consequences if not addressed accordingly. Patient presentation varies with the type and timing of the leakage and the patient’s systemic inflammatory response.

Gastric leakages could be due to the creation of a long and narrow tube to maintain the pylorus, which surely increases the intraluminal pressure (intragastric hypertension), localized ischaemia related to impaired vascularization of the gastroepiploic arteries, or short ligation of the gastric vessel during detachment from the greater curvature [22, 23]. The use of routine intraoperative methylene blue for leak testing is an ongoing discussion as this method may be helpful in identifying suture line breaks (but cannot absolutely recognize) any areas at increased risk for subsequent leakage [24–26].

ICG fluorescence angiography facilitates evaluation of the blood supply during surgery, and is considered an inexpensive and feasible method to establish vascularity in the desired area [27, 28].

In our study, one leakage was recorded in an ICG-tested LSG despite achieving adequate underlying cause of the leak is multifactorial, and leakages are not only due to poor vascularization of the gastric tract.

The group leakage rate was 1.2%: this rate was lower to that of the nontested patients, but to better understand this finding, we need to increase the number of LSGs with follow-up data to obtain significant values.

Moreover, our main goal was to evaluate if this method could adequately predict ischaemia along the staple line of the stomach and help prevent gastric leakage; currently, this has not been confirmed from our preliminary results (the single case in which leakage occurred showed adequate perfusion with the ICG test).

The ICG test has been fully and absolutely approved in most laparoscopic procedures [29], but there is still little literature on its usefulness within bariatric procedures.

Therefore, our aim was to evaluate whether intraoperative ICG fluorescence angiography could help identify possible ischaemic areas [30, 31].

From the initial data, we did not find any segmental areas with ischaemia along the greater curvature, focusing mainly on the area near the angle of His, despite one patient developing gastric leakage on the fifth postoperative day.

From our study, we observed a lower leakage rate in the group tested with ICG, but these are preliminary data, and studies with larger numbers of patients are needed. This study represents a valid starting point for further research to collect a greater amount of data and obtain statistically significant results.

## Conclusions

Despite the lower leak ratio of patients undergoing the ICG test, the occurrence of gastric fistula could not be ruled out; in fact, all the patients who underwent ICG testing during LSG showed optimal perfusion. These data led us to believe that multiple factors are related to the pathophysiology and incidence of gastric fistula in the context of LSG operations.

Therefore, intraoperative ICG testing may be helpful in determining which patients are at an increased risk for leakage and if adjunctive measures are needed intraoperatively, but further testing is required to determine if ICG will predict leakage due to ischaemia.

## Data Availability

All data generated or analysed during this study are included in this published article.
